# *Fagopyrum esculentum* Alters Its Root Exudation after *Amaranthus retroflexus* Recognition and Suppresses Weed Growth

**DOI:** 10.3389/fpls.2018.00050

**Published:** 2018-01-31

**Authors:** Aurélie Gfeller, Gaétan Glauser, Clément Etter, Constant Signarbieux, Judith Wirth

**Affiliations:** ^1^Herbology in Field Crops and Viticulture, Plant Production Systems, Agroscope, Nyon, Switzerland; ^2^Neuchâtel Platform of Analytical Chemistry, University of Neuchâtel, Neuchâtel, Switzerland; ^3^Laboratory of Ecological Systems ECOS, School of Architecture, Civil and Environmental Engineering ENAC, École Polytechnique Fédérale de Lausanne (EPFL), Lausanne, Switzerland

**Keywords:** allelopathy, buckwheat, heterospecific recognition, pigweed, root exudation, weed suppression

## Abstract

Weed control by crops through growth suppressive root exudates is a promising alternative to herbicides. Buckwheat (*Fagopyrum esculentum*) is known for its weed suppression and redroot pigweed (*Amaranthus retroflexus*) control is probably partly due to allelopathic root exudates. This work studies whether other weeds are also suppressed by buckwheat and if the presence of weeds is necessary to induce growth repression. Buckwheat and different weeds were co-cultivated in soil, separating roots by a mesh allowing to study effects due to diffusion. Buckwheat suppressed growth of pigweed, goosefoot and barnyard grass by 53, 42, and 77% respectively without physical root interactions, probably through allelopathic compounds. Root exudates were obtained from sand cultures of buckwheat (BK), pigweed (P), and a buckwheat/pigweed mixed culture (BK-P). BK-P root exudates inhibited pigweed root growth by 49%. Characterization of root exudates by UHPLC-HRMS and principal component analysis revealed that BK and BK-P had a different metabolic profile suggesting that buckwheat changes its root exudation in the presence of pigweed indicating heterospecific recognition. Among the 15 different markers, which were more abundant in BK-P, tryptophan was identified and four others were tentatively identified. Our findings might contribute to the selection of crops with weed suppressive effects.

## Introduction

Plants perceive their above- and belowground environment by gathering information on the availability of the resources light, nutrients and water or chemical cues such as volatile compounds, leachates and root exudates (Weston and Mathesius, 2013; Fernandez et al., 2016). Roots exude a complex range of compounds into the surrounding soil, like ions, inorganic acids, free oxygen, water and different carbon-based compounds such as amino acids, polysaccharides, and others (Bais et al., 2006; De-la-Peña and Loyola-Vargas, 2014). Root exudates are important in mediating interactions with microbes and neighboring plants (Bais et al., 2004, 2006; Weir et al., 2004; Broeckling et al., 2008) and their production and release can be constitutive or activated in response to abiotic and biotic stress (De Albuquerque et al., 2011; Weston et al., 2012; Latif et al., 2017). There is growing evidence that plants recognize and respond to heterospecific neighboring plants and this might lead to competitive advantages (Broz et al., 2010; Depuydt, 2014). For example, after recognition of neighboring weeds, rice (*Oryza sativa*) and sorghum (*Sorghum bicolor*) change root exudation through the increased production of allelopathic root exudates such as momilactone and sorgoleone respectively (Zhao et al., 2005; Dayan, 2006; Kato-Noguchi and Peters, 2013). In a context of low herbicide use, plant breeders have an interest in the selection of allelopathic crops that are able to recognize and effectively suppress weeds. Therefore, it is important to characterize to which extend neighboring plants interact.

The overall goal of our research is to develop diversified cropping systems without the use of herbicides. One strategy is the use of weed suppressive cover crops like for example buckwheat (*Fagopyrum esculentum*), which is known to suppress weeds in the field (Tominaga and Uezu, 1995; Creamer and Baldwin, 2000; Kalinova, 2004; Gfeller et al., 2018). Several studies have been done on buckwheat allelopathy (Tsuzuki et al., 1987; Kalinova et al., 2005, 2007; Golisz et al., 2007; Kato-Noguchi et al., 2007; Tin et al., 2009). However, the mechanisms responsible for the weed suppressive effect have not yet been clearly identified (Falquet et al., 2015). We have previously shown that redroot pigweed (*Amaranthus retroflexus*) aboveground biomass was significantly reduced by buckwheat independently of resource competition and we concluded that allelopathic root interactions were promoting this growth inhibition (Gfeller et al., 2018). By considering the theory on costs of plant defense in stressful environments, predicting that costs should increase when competition is intense (Siemens et al., 2002), we further hypothesized that buckwheat changes its root exudation profile in the presence of weeds in order to suppress their growth. To elucidate this, we studied the impact of direct and indirect root interactions between buckwheat and three different annual summer weeds on different growth parameters in field soil. Moreover, we studied buckwheat root exudates in the presence and absence of pigweed obtained from sand cultures. Based on the complementary approach with two distinct sets of experiments, we tested three hypotheses (H). H1: Roots of buckwheat and different weeds do not have to physically interact to induce weed growth repression. H2: Buckwheat root exudates induce pigweed growth repression, when both plant species grow next to each other. H3: The presence of pigweed induces changes in buckwheat root exudation.

## Materials and Methods

### Plant Material Used

Buckwheat (*Fagopyrum esculentum* Moench, variety Lileja; Polygonaceae) was provided by the cooperative FENACO (Switzerland). For the weed species, redroot pigweed (*Amaranthus retroflexus*; Amaranthaceae) and goosefoot (*Chenopodium album* L., Amaranthaceae) seeds were obtained from Herbiseed (Twyford, GB), while barnyard grass (*Echinochloa crus-galli* L. P. Beauv., Poaceae) was collected in a field close to our research institute in Switzerland (46°23′56′ N, 6°13′54′ E).

### Pot Trials with Soil

**FIGURE 1:**
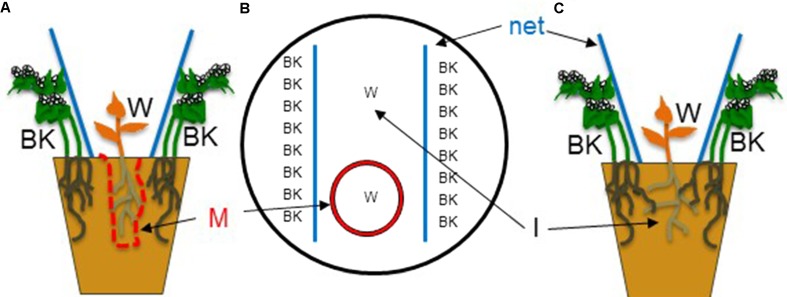
Experimental design of the pot trial. Eight buckwheat (BK) plants were grown on the two outer sides of each pot. In the center, one weed (W) was grown with direct root interactions with BK plants (I) and one with no direct root interactions between the two species within a mesh bag (M). Two nets (net) pushing aside BK foliage and strongly reducing shading were placed between the BK and weed plants. Side view of the condition with the mesh bag **(A)**. Looking at a pot from the top **(B)**. Side view of the condition where roots of the two species are interacting **(C)**. Reprinted and adapted from Gfeller et al. (2018) with permission from Elsevier.

The method used is a development of previously described protocols (Falquet et al., 2014; Gfeller et al., 2018). In order to increase germination rate, pigweed seeds were heat treated during 1 h at 55°C, whereas barnyard grass and goosefoot seeds were soaked in KNO_3_ (101 mg/L) for 4 h. Buckwheat seeds did not require pre-treatment. Four days before the start of the experiment, all seeds were placed on humidified Whatman paper under dark conditions at 24°C for germination. On day zero, all pots (3 L) were filled with field soil previously heated at 60°C for 6 h and sieved to 3 mm. Two lines of eight homogeneously germinated buckwheat seedlings were placed at a depth of 2 cm in parallel lines close to the outer sides of each pot containing buckwheat (conditions BK) (**Figure 1**). No buckwheat was sown in the control pots (conditions C). In the center of each pot (BK and C) three pre-germinated seeds of goosefoot, barnyard grass or pigweed were placed in two different sowing areas: on the surface of the soil (I = direct root interactions,) and within a mesh bag (Sefar Nitex 30 μm, 12 cm × 22 cm) filled with 220 g of the same soil (M = no direct root interactions, mesh bag). The mesh allows exchange of solutes but does not allow roots to penetrate. An overlay of 3 mm sieved soil (1.5 mm) was placed over the seeds. Pots were placed in a phytotron (14 h light period, 24°C/18°C day/night, 70% Humidity) and daily watered for 1 week. Four conditions were tested: (1) weeds in control pots without buckwheat (C-I), (2) weeds growing inside a mesh bag in control pots without buckwheat (C-M), (3) weeds and buckwheat growing together with direct root interactions of the two species (BK-I) and (4) weeds and buckwheat growing together without direct root interactions of the two species due to the presence of the mesh (BK-M). Seven pots per condition were used. On day five, each weed species was reduced to one individual plant per sowing area. From day five onward, three times per week during the whole experiment weed height measurements were taken. Plant height was measured from the soil surface to the insertion of the youngest leaf for the two dicotyledonous plants goosefoot and pigweed, and to the top of the highest leaf for the monocotyledon barnyard grass. From day 8 onward, plants were daily watered with a nutrient solution (Wuxal Profi, Maag, N 100 g/L, P_2_O_5_ 100 g/L, K_2_O 75 g/L, 0.5%) below the leaves to avoid foliar leaching. On day 10, the buckwheat canopy was wedged open with two vertical nets in order to allow all individual weed plants to receive the same amount of light. On day 28, prior to the harvest of the aerial biomass of all plants, the tiller number per plant of barnyard grass was counted. In addition, the roots of the three weeds from the mesh compartments were harvested. Roots from buckwheat and from the weeds directly interacting with each other were not collected as they were tangled together and could not be separated. After drying at 65°C during 72 h dry weight (DW) was determined. The whole experiment was repeated three times.

### Chlorophyll *a* Fluorescence Measurements in Pot Trials

Chlorophyll *a* fluorescence was recorded for the three weed species on days 17 and 27 with a portable pulse amplitude fluorometer (PAM-2500, Heinz Walz GmbH, Effeltrich, Germany). F_v_/F_m_ as the maximum quantum yield of photosystem II was determined and calculated according to Maxwell and Johnson (2000). Once the leaves were dark-adapted for 30 min using the leaf-clip holder 2030B (Heinz Walz GmbH, Effeltrich, Germany), F_v_/F_m_ was recorded via the fiber optic of the PAM-2500. F_v_/F_m_ was used here as a stress indicator and gave information on the non-stomatal limitation of photosynthesis (Signarbieux and Feller, 2011) and more generally about plant health and vigor (Adams Iii et al., 2004).

### Soil Nutrient Measurements in Pot Trials

In the last replication of the experiment, soil samples were taken in three randomly chosen pots at harvest day from both conditions (I and M) for pots containing goosefoot and barnyard grass and for control pots and immediately frozen at -20°C. Plant available nutrients (NO_3_, P, K, Ca, and Mg) in the soil solution were determined by water extraction with a 1:10 soil-water ratio by an external laboratory (Ibu, Thun, Switzerland) according to reference methods (Myers and Paul, 1968; Agroscope, 1996).

### Plant Cultivation for Root Exudation Experiments with Glass Sand

Buckwheat and pigweed seeds used in this additional experiment were not pre-treated and not pre-germinated. Cleaned and autoclaved glass sand (75 g, Glas-Sand für Sandfilteranlagen, Waterman GmbH, Wendlingen, Germany) was disposed in transparent plastic boxes (13 cm × 10.5 cm × 10 cm) and humidified with 20 mL of 1x Hoagland solution. Twenty five buckwheat seeds were placed on the surface of the glass sand, covered with 40 g of glass sand and humidified with 10 mL Hoagland solutions. Pigweed seeds (0.08 g) were placed on the surface of the glass sand. A second box was used as a lid and the boxes were sealed with surgical tape. Boxes were placed in the phytotron (14 h light period, 24°C/18°C day/night) for 11 days. Four conditions were tested: (1) control boxes with sand only, no plants (C), (2) only pigweed plants (P), (3) only buckwheat plants (BK) and (4) a buckwheat/pigweed mixed culture (BK-P). Three boxes per condition were used.

### Extraction of Glass Sand Collected Root Exudates

On day 11, buckwheat plants were harvested by pulling them out of the sand in conditions BK and BK-P. Roots with glass sand attached on them, were cut and disposed in a 250 mL flat bottom flask. For conditions C and P, 50 g of glass sand were taken. Fifty milliliter of methanol (Sigma, Switzerland) was added to each flask. Flasks were stirred for 1 h at 220 rpm. The liquid was recovered, filtrated through a gauze and centrifuged at 5000 rpm for 5 min. The supernatant was passed through a 0.45 μm cellulose acetate filter and evaporated in a freeze dryer (GeneVac) until around 0.5 mL was left. Samples were then completely evaporated under nitrogen flux. They were then resuspended in 50 μL (for biological tests) and 200 μL (for root exudates analysis) of methanol, vortexed and sonicated. Then they were centrifuged at 9000 rpm for 5 min, and the supernatant was recovered and conserved at -20°C. Three and two independent experiments were performed for biological tests and for root exudates analysis respectively.

### Biological Tests with Root Exudate Extracts

Root exudate extracts were diluted with tap water to 1%. Whatman paper in a petri dish (12 cm × 12 cm) was humidified with 4 mL of diluted root exudates. Two rows of 12 pigweed seeds were disposed in each petri dish which was sealed with parafilm. Pigweed was grown vertically in the dark and at room temperature for 5 days. Germination rate, root and hypocotyl length were determined after 5 days.

### Metabolomic Analysis of Root Exudate Extracts

Metabolite profiling was performed by ultrahigh pressure liquid chromatography-high resolution mass spectrometry (UHPLC-HRMS) using an Acquity UPLC (Waters) coupled to both an eλ PDA detector and a Synapt G2 QTof mass spectrometer (MS). The separation was performed using an Acquity UPLC BEH C18 column (50 mm × 2.1 mm) in gradient mode at a flow rate of 0.4 mL/min. Mobile phase A was water + formic acid 0.05% and mobile phase B was acetonitrile + 0.05% formic acid. The following program was applied: 5–30% B in 6 min, 30–100% B in 2 min, holding at 100% B for 2 min, requilibrating at 5% B for 1.5 min. The temperature of the column was maintained at 25°C. The injection volume was 2.5 μL. The column void time was 0.26 min. PDA detection was achieved at a frequency of 20 Hz from 190 to 600 nm with a resolution of 1.2 nm. The QTOF MS was operated in negative electrospray ionization over a mass range of 85–1200 Da. The MS^E^ mode was used, in which the collision cell of the mass spectrometer alternatively switches between low and high collision energies while the quadrupole serves as an ion guide. Source parameters were as follows: capillary voltage -2.0 kV, cone voltage -25 V, desolvation temperature and gas flow 350°C and 800 L/h, respectively, source temperature 120°C. Accurate mass measurements were provided by infusing a calibration solution of leucine-enkephalin throughout the run using the Lockspray probe. LC-MS data were processed by Markerlynx XS (Waters) to generate a list of markers characterized by their retention time (RT) and their mass to charge ratio (m/z). Finally, we attempted to identify markers by combining determination of elemental compositions, mass fragment characteristics and search in online databases.

### Statistical Analysis

All statistical analysis were conducted in R environment with the version 3.2.2 (R Developmental Core Team, 2014).

#### Pot Trial

**FIGURE 2:**
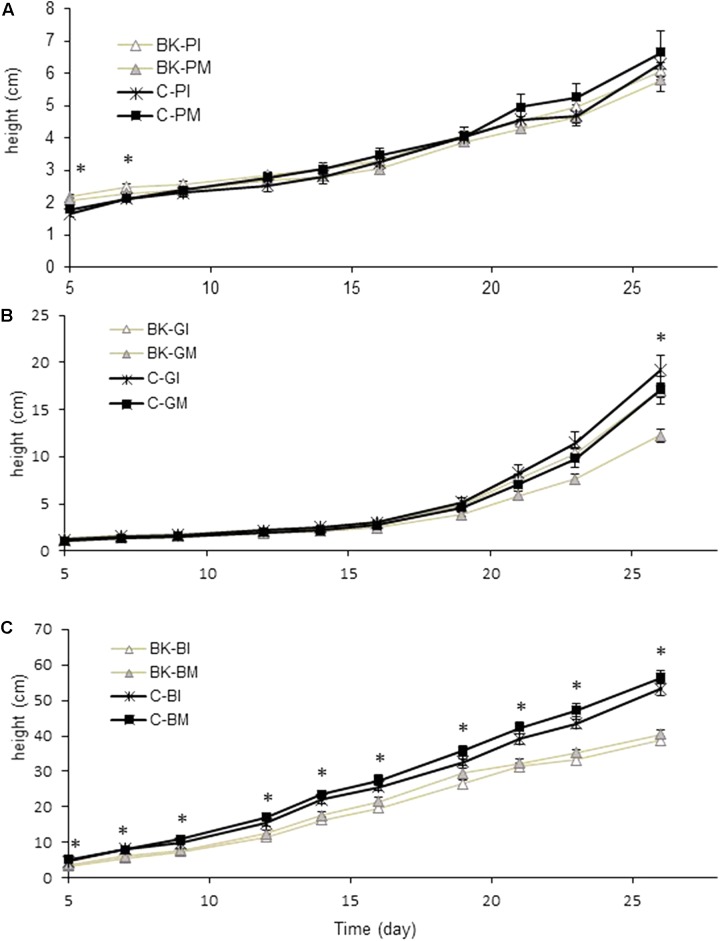
Weed height measurements in the pot trial. Weed height was regularly measured throughout the experiment until day 28. Pigweed **(A)** and goosefoot **(B)** height was measured from the soil surface to the insertion of the youngest leaf. Barnyard grass **(C)** height was measured from the soil surface to the top of the highest leaf. BK, buckwheat; P, pigweed; G, goosefoot; B, barnyard grass; C, control; I, direct root interactions; M, no direct root interactions, mesh bag. One factor ANOVA (C vs. BK) was performed at each data point. ^∗^*p*-value < 0.05, *N* = 14.

**FIGURE 3:**
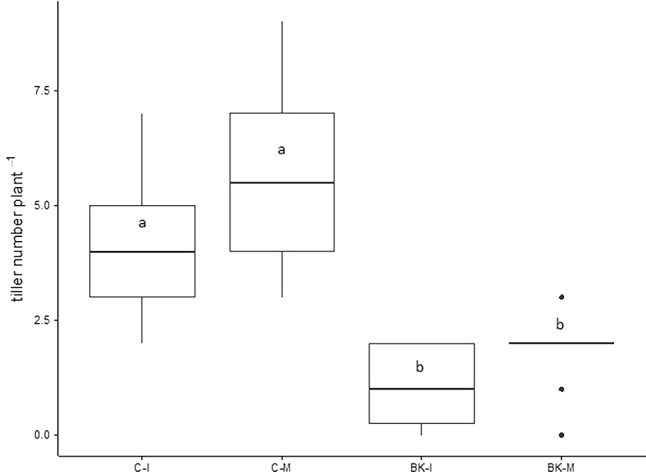
Tiller number per barnyard grass in the pot trial. On day 28 of the pot trial tiller number of barnyard grass was counted. BK, buckwheat; C, control; I, direct root interactions; M, no direct root interactions, mesh bag. Tukey’s HSD, *p*-value < 0.05, *N* = 21.

**FIGURE 4:**
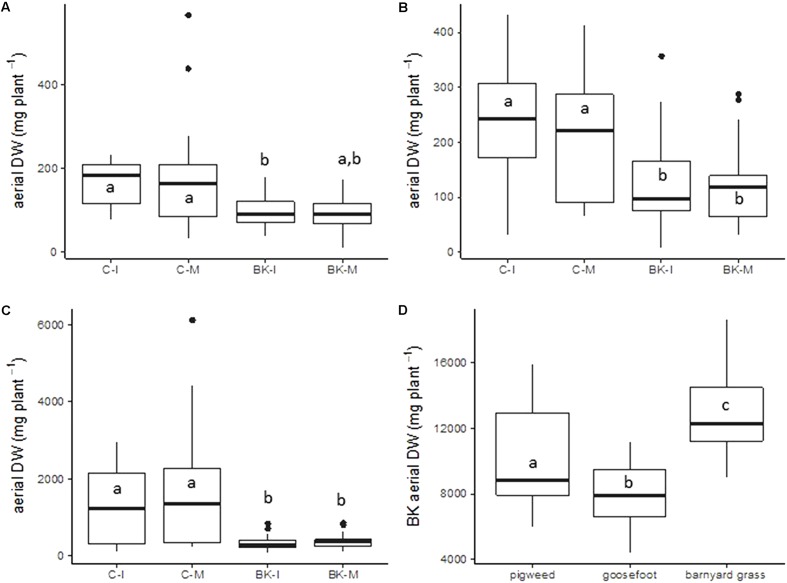
Dry weight of weed aerial biomass in the pot trial. On day 28 of the pot trial aerial biomass of the weeds pigweed **(A)**, goosefoot **(B)** and barnyard grass **(C)** and of buckwheat **(D)** was harvested and subsequently dried to obtain aerial dry weight (DW). BK, buckwheat; C, control; I, direct root interactions; M, no direct root interactions, mesh bag. Wilcoxon test, *p*-value < 0.05, *N* = 14 for pigweed **(A)**, *N* = 21 for goosefoot **(B)**, and barnyard grass **(C)**. Tukey’s HSD, *p*-value < 0.05 for buckwheat **(D)**.

**FIGURE 5:**
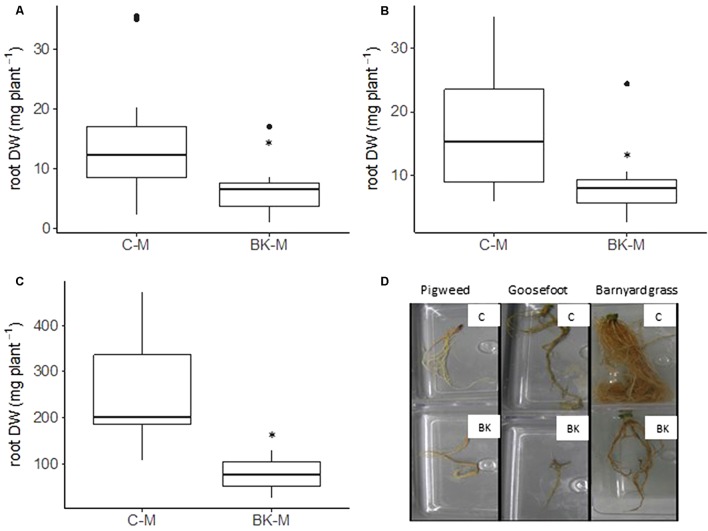
Dry weight of root biomass in the pot trial. On day 28 of the pot trial root biomass from the mesh compartments of pigweed **(A)**, goosefoot **(B)** and barnyard grass **(C)** was harvested and subsequently dried to obtain dry weight (DW). Photos of the roots on harvest day **(D)**. BK, buckwheat; C, control; M, no direct root interactions, mesh bag. Student’s *t*-test, *p*-value < 0.05, *N* = 14.

**FIGURE 6:**
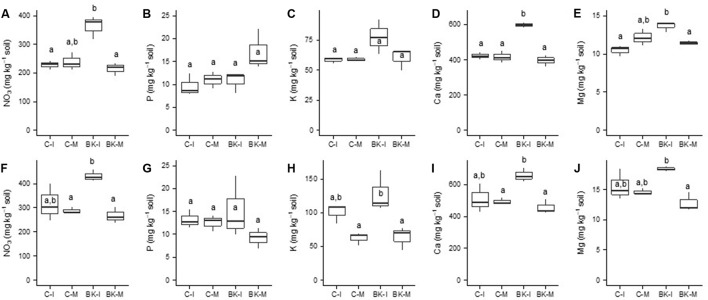
Plant available nutrients in the pot trial. On day 28 of the pot trial soil samples were taken in the mesh compartments (M) and in zones where buckwheat and weed roots were directly interacting (I) in pots with barnyard grass **(A–E)** and goosefoot **(F–J)**. Plant available nutrients were determined by water extraction with a 1:10 soil-water ratio. Nitrate NO_3_ **(A,F)**, Phosphorus P **(B,G)**, Potassium K **(C,H)**, Calcium Ca **(D,I)**, Magnesium Mg **(E,J)**, BK, buckwheat; C, control; I, direct root interactions; M, no direct root interactions, mesh bag. Tukey’s HSD, *p*-value < 0.05, *N* = 3.

For weed height, a one factor ANOVA was applied at each data point (**Figure 2**). A Tukey’s HSD test was applied on data sets of barnyard grass tiller number (**Figure 3**), buckwheat biomass (**Figure 4D**) and plant available nutrients (**Figure 6**). For aerial weed biomass a pairwise Wilcoxon rank sum test with “Holm” correction for multiple testing was performed (**Figures 4A–C**) and for root biomass and a two-tailed paired Student’s *t*-test was done (**Figure 5**).

#### Root Exudation Experiments with Glass Sand

**FIGURE 7:**
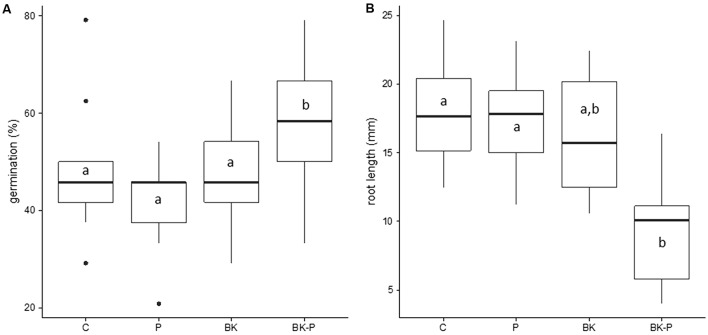
Pigweed germination and root length in the presence of different root exudates from sand cultures. Percentage germination of pigweed seeds **(A)** and pigweed root length **(B)** in petri dishes 5 days after sowing in the presence of root exudates obtained from 11 days old sand cultures of control boxes without plants (C), with pigweed (P), with buckwheat (BK) and with a buckwheat/pigweed mixed culture (BK-P). Tukey’s HSD, *p*-value < 0.05, *N* = 9.

**FIGURE 8:**
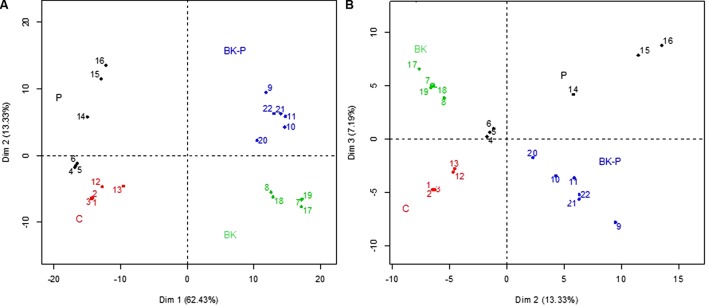
Principal component analysis (PCA) on markers obtained from the different root exudates from sand culture and separated by UHPLC-HRMS. PCA score plots of dimension (Dim) 1 and 2 **(A)** and dimension 2 and 3 **(B)**. Root exudates were obtained from 11 days old sand cultures of control boxes without plants (C), with pigweed (P), with buckwheat (BK) and with a buckwheat/pigweed mixed culture (BK-P), *N* = 6.

**FIGURE 9:**
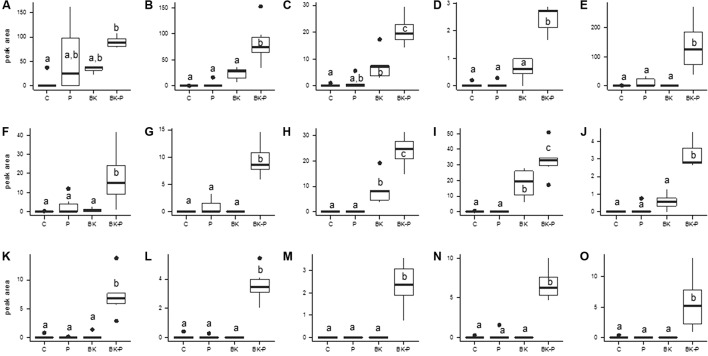
Markers from the different root exudates from sand culture which were more abundant in the buckwheat/pigweed mixed culture. Root exudates obtained from 11 days old sand cultures of control boxes without plants (C), with pigweed (P), with buckwheat (BK) and with a buckwheat/pigweed mixed culture (BK-P) and analyzed by UHPLC-HRMS. A selection of markers were identified as L-Tryptophan **(A)** and tentatively identified as Fructose-Leucine or Fructose-Isoleucine **(B)**, Fructose-Phenylalanine **(C)**, C_13_H_19_NO_7_ **(D)**, C_14_H_20_O_7_ **(E)**, *N*-acetyl glutamic acid methyl ester **(F)**, a sulfur-containing compound C_14_H_34_O_10_S **(G)**, C_25_H_33_N_3_O_7_ **(H)**, C_12_H_13_NO_6_ **(I)** and C_5_H_10_O_3_ **(J)**. The following markers are unidentified with the respective retention time and m/z of 1.696; 365.135 **(K)**, 0.940; 401.082 **(L)**, 0.958; 660.209 **(M)**, 2.923; 305.106 **(N)**, and 6.919; 297.098 **(O)**. Tukey’s HSD, *p*-value < 0.05, *N* = 6.

For germination rate (**Figure 7A**), root and hypocotyl length (**Figure 7B**), Tukey’s HSD test was performed to compare the different treatments. ANOVA was conducted with the values of the peak areas of all markers obtained from the metabolomic analysis and the variables with a *p*-value < 0.01 were selected to perform a principal component analysis (PCA) with the R package Factomine R with default parameters (**Figure 8**). We tried to identify the most important variables that explain the variations between the conditions BK and BK-P in our data by looking at the variable vector map, applying a *t*-test between BK and BK-P and by choosing the markers with the highest peak areas (**Figure 9**). Differences between treatments were tested with Tukey’s HSD test.

## Results

### Effect of the Presence of Buckwheat on Weed Growth Parameters

Plant height of the three weeds was differently affected by the presence of buckwheat (**Figure 2**). Pigweed plants growing in association with buckwheat (BK-PI and BK-PM) significantly differed from pigweed control plants (C-PI and C-M) at days 5 and 7 of the experiment (**Figure 2A**). Goosefoot plant height in the presence of buckwheat (BK-GI and BK-GM) was significantly smaller than the control (C-GI and C-GM) after 26 days (**Figure 2B**). Barnyard grass height was reduced all over the experiment when grown together with buckwheat (BK-BI and BK-BM) (**Figure 2C**). Barnyard grass tillering was significantly reduced in the presence of buckwheat (BK-I and BK-M) after 28 days (**Figure 3**).

Weed aerial biomass after 28 days was significantly reduced for the three weeds when grown together with buckwheat (BK-I and BK-M) (*p*-value < 0.05) (**Figures 4A–C**). Direct root interactions between the two species (BK-I vs. C-I) reduced aboveground biomass of the Amaranthaceae pigweed and goosefoot by 41 and 48% and the Poaceae barnyard grass by 75% respectively. Indirect root interactions (BK-M vs. C-M) provoked a reduction of growth by 53, 42, and 77% for pigweed, goosefoot and barnyard grass. Weed aerial biomass in the two experimental conditions BK-I and BK-M were the same, despite partly significant differences in soil nutrient contents. We therefore assume that nutrient contents did not have an impact on the observed weed growth reduction as already previously suggested (Falquet et al., 2014; Gfeller et al., 2018). Weeds growing in the control pots with a large soil volume (C-I) and within the meshes with a reduced soil volume (C-M), developed the same aboveground biomass. This observation shows that the reduced soil volume inside the mesh did not negatively influence weed aerial biomass. Buckwheat biomass after 28 days was different depending on the weed association (**Figure 4D**). Buckwheat growth was the highest with barnyard grass and the lowest with goosefoot. Roots from all weeds within the mesh in association with buckwheat (BK-M) were significantly smaller than roots from weeds grown in control pots (C-M) (**Figure 5**) (*p*-value < 0.05). Reduced growth was of 56, 51, and 70% for pigweed, goosefoot, and barnyard grass respectively.

### Chlorophyll *a* Fluorescence

Additionally, to understand whether differences in growth reduction could be due to metabolic down-regulation of photosynthesis (Signarbieux and Feller, 2011), we measured maximum quantum yield of photosystem II (F_v_/F_m_) on the three weeds species at days 17 and 27 in the pot trial. F_v_/F_m_ did not show significant differences between the four growth conditions and was highly consistent with values between 0.75 and 0.85. This indicates that the photosynthetic electron transport was intact and that no apparent non-stomatal regulation processes could be linked the observed species differences in growth (data not shown).

### Nutrient Supply in Pot Trials

Plant available nutrient contents (NO_3_, P, K, Ca, and Mg) in the control pots (C-I and C-M) and in the meshes of pots with buckwheat (BK-M) were not significantly different for both weeds (**Figure 6**). All plant available nutrients measured at the end of the experiment were high enough to exclude nutrient deficiencies. Nutrient concentrations in the soil outside the meshes (BK-I) were partly higher than in the other conditions. Soil nitrate (**Figures 6A,F**), calcium (**Figures 6D,I**) and magnesium (**Figures 6E,J**) contents were significantly higher when barnyard grass and goosefoot roots were interacting with buckwheat (BK-I). Soil potassium (**Figure 6H**) content was only higher when goosefoot roots were interacting with buckwheat (BK-I).

### Activity of Root Exudate Extracts on Pigweed Germination and Growth

Root exudate extracts obtained from 11 days old sand grown cultures of pigweed (P), buckwheat (BK) and a buckwheat/pigweed mixed culture (BK-P) were used for germination and growth assays with pigweed in petri dishes. After 5 days, root exudate extracts from BK-P caused a higher germination rate for pigweed (**Figure 7A**) and strongly reduced pigweed root growth by 49% compared to the control (C) (**Figure 7B**). Pigweed hypocotyl growth was not effected (data not shown).

### Metabolomic Analysis of Root Exudate Extracts

In total, a list of 3506 different markers was generated after analysis of root exudate extracts by UHPLC-HRMS. PCA was applied to discriminate among the different experimental conditions (**Figure 8**). The main principal component (PC) to differentiate between samples, i.e., PC1, accounted for 62.43% of the variance, with the experimental conditions C and P located to the left of the vertical line representing PC1 (negative PC1 values), and the conditions BK and BK-P positioned to the right of the PC1 axis (**Figure 8A**). PC2, accounted for 13.33% of the variation with P and BK-P clustered at the positive values and control and buckwheat at the negative values. PC3 accounted for 7.19% of the variance (**Figure 8B**).

However in most cases, full identification of the most promising compounds was not possible due to a lack of information in the databases. This is not really surprising as buckwheat, a Polygonaceae, and pigweed, an Amaranthaceae, are not common plant models. We chose 15 markers that show interesting and highly significant differences between the conditions based on the significance level, the best projection of the PCA vector map in the dimension 2 and the total abundance. Apart from L-tryptophan, which was confirmed by a standard (**Figure 9A**), the following compounds have to be considered as tentatively identified: fructose-leucine or fructose-isoleucine (**Figure 9B**), Fructose-Phenylalanine (**Figure 9C**), C_13_H_19_NO_7_ (**Figure 9D**), C_14_H_20_O_7_ (**Figure 9E**), *N*-acetyl glutamic acid methyl ester (**Figure 9F**), a sulfur-containing compound C_14_H_34_O_10_S (**Figure 9G**), C_25_H_33_N_3_O_7_ (**Figure 9H**), C_12_H_13_NO_6_ (**Figure 9I**), and C_5_H_10_O_3_ (**Figure 9J**). The remaining markers are unidentified with the respective retention time and m/z of 1.696; 365.135 (**Figure 9K**), 0.940; 401.082 (**Figure 9L**), 0.958; 660.209 (**Figure 9M**), 2.923; 305.106 (**Figure 9N**) and 6.919; 297.098 (**Figure 9O**). Some markers are present in root exudate extracts from BK-P and not present or only as traces in the other conditions (**Figures 9K–O**), some are present in the BK extracts but more abundant in BK-P (**Figure 9B–D,H–J**) and some are present in the P extracts and more abundant in BK-P (**Figures 9E–G**) and finally tryptophan was present in BK and P extracts, but more abundant in BK-P.

## Discussion

### Pigweed, Goosefoot, and Barnyard Grass Growth Suppression by Buckwheat

Our experimental design of the pot trial allowed to study the effect of root exudates of a donor plant on the growth of a target plant under homogenous conditions in soil. The presence of buckwheat strongly reduced weed growth, implicating that the observed growth reduction is a direct consequence of the presence of buckwheat and not of resource competition. Indeed, we consider that light availability was equal for all weeds and water and nutrients were supplied regularly in sufficient amounts throughout the experiment. The maximum efficiency of photosystem II (F_v_/F_m_) for all weeds was similar and showed healthy and intact photosystem II (Butler, 1978; Demmig and Björkman, 1987; Genty et al., 1989). Hence, biomass reduction observed for the weed species is most likely not linked to any non-stomatal down-regulation of photosynthesis.

As recently reported for pigweed (Gfeller et al., 2018), a significant reduction in aerial biomass through direct and indirect root interactions with buckwheat could also be observed for barnyard grass and goosefoot. This indicates that weed suppression by buckwheat is not species specific for pigweed but a more general phenomenon concerning weed species from different families. We were furthermore able to show that root growth of the three tested weed species was also strongly reduced by the presence of buckwheat. Growth impairment was strongest for barnyard grass, where plant height, a major determinant of a plant’s ability to compete for light, as well as tiller number were also strongly negatively affected. Because plant height and traits such as leaf mass fraction, leaf area ratio, leaf mass per area and canopy area are correlated (Falster and Westoby, 2003; Moles et al., 2009) and because plant height influences seed mass, time to reproduction and the number of seeds (Moles and Leishman, 2008; Moles et al., 2009), we assume that the presence of buckwheat might affect the successful propagation of barnyard grass to a larger extent.

Possible explanations for the observed effects could be the emission of volatile organic compounds, modification of soil microorganisms and/or allelopathic buckwheat root exudates. It has been shown that root exudates influence soil microorganisms, which then affect plant growth as for example secondary metabolites from *Empetrum hermaphroditum* which inhibit symbiotic associations between *Pinus sylvestris* trees and mycorrhiza fungi, leading to reduced nitrogen uptake by Pinus (Nilsson et al., 1993; Bais et al., 2006). Moreover, root exudates may change activities of allelopathic bacteria in the rhizosphere of non-host species (Abbas et al., 2017). However, in this study, we decided to focus on root exudates and hypothesize that compounds exuded by buckwheat roots are responsible for the observed effects, as suggested by previous studies (Kalinova et al., 2007; Kato-Noguchi et al., 2007; Gfeller et al., 2018). The same growth repressive effects could be observed for the three weeds grown in the two distinct compartments BK-I and BK-M, indicating that roots of buckwheat and different weeds do not have to physically interact to induce weed growth repression (Hypothesis 1). However, we cannot exclude that root hairs from weeds inside the mesh and surrounding buckwheat plants partially touched as in order to restrict root hair growth a mesh pore size of 7 μm is necessary (Wenzel et al., 2001). We hypothesize that one or several solutes from buckwheat root exudates passed into the mesh and caused the observed weed growth repression in the BK-M conditions.

### Buckwheat Changes Its Root Exudation Profile after Heterospecific Neighbor Recognition

We have seen that weed growth was suppressed due to the presence of buckwheat. What if buckwheat recognizes the presence of the weeds and subsequently changes its root exudation profile in order to impair their growth? In order to answer this question, we studied root exudates from buckwheat growing alone or together with pigweed. Pigweed was chosen as a test weed due to results from previous studies (Gfeller et al., 2018). We could show for the first time that pigweed germination increased in the presence of root exudate extracts from a co-cultivation of buckwheat and pigweed but not when buckwheat was cultivated alone. It is known that strigolactones are secreted from roots into the soil, stimulating seed germination of parasitic weeds from the Orobanchaceae family (De Cuyper and Goormachtig, 2017). Furthermore, no other studies showing positive effects of root exudates on seed germination could be found in the literature. The same root exudate extracts causing increased pigweed germination strongly inhibited pigweed root growth, demonstrating that buckwheat root exudates induce pigweed growth repression, when both plant species grow next to each other (Hypothesis 2). Pigweed growth repression by buckwheat root exudates could be demonstrated in soil and with root exudate extracts from plants cultivated in glass sand. This suggests that metabolite composition of root exudates, which depend on the biotic and abiotic environment to which roots are subjected (van Dam and Bouwmeester, 2016), was not influenced by the growth systems and indicates that soil microorganisms were not implicated directly in the observed results as they were absent in the methanolic root exudate extracts applied in the petri dish experiments. Glass sand was autoclaved, but buckwheat and pigweed seeds were not surface sterilized which does not exclude that root exudates could have been partly modified by microorganisms associated with pigweed and buckwheat seeds. From our results we conclude that one or several soluble compounds present in root exudate extracts of BK-P provoked the observed effects, rather than any information communicated by direct root contact between the two species. In the PCA analysis root exudate extract composition of BK-P separated from the other experimental conditions, showing that root exudate extract composition was different when both species were co-cultivated. This supports the third hypothesis that pigweed recognition by buckwheat induces changes in buckwheat root exudation profile. This newly discovered mechanism is subtle as on one side pigweed germination is stimulated, which favors pigweed success, and on the other side pigweed root growth is inhibited, which favors buckwheat success. In a second step we have to elucidate whether the same compounds are responsible for the two distinct effects.

It has been shown that Arabidopsis plants actively change root allocation patterns under inter- or intraspecific competition which is probably due to specific exudate cues (Schmid et al., 2013; van Dam and Bouwmeester, 2016). We suggest that in our case heterospecific neighbor recognition occurred. Compounds might have been liberated from roots of the two co-cultivated species inducing a chemical cross-talk, which resulted in a modified buckwheat root exudation profile leading to the observed effects. Our observations are similar to the work of Dayan (2006), who showed that sorgoleone production by sorghum (*Sorghum bicolor*) was induced by extracts of velvetleaf root and of Kato-Noguchi (2011) who suggested that rice may respond to the presence of neighboring barnyard grass by sensing the components in barnyard grass root exudates and increasing allelopathic activity by producing elevated concentrations of momilactone B. However, perception of exudate compounds by plants is still an unknown mechanism (van Dam and Bouwmeester, 2016).

### Potential Allelochemicals Implicated in Pigweed Growth Suppression

We could show that buckwheat changes its root exudation profile when pigweed is present since 600 markers were more abundant when both plants are growing next to each other (*t*-test between BK and BK-P; not shown). From the 15 most abundant molecules that statistically responded to the different treatments 4 molecules could at least be tentatively identified as one amino-acid, one amino acid derivate and two fructose-amino acids. Not surprisingly, amino acids and sugars are abundant compounds of root exudates (van Dam and Bouwmeester, 2016). The amino acid, L-Tryptophan is a known allelochemical from *Prosopis juliflora* (Nakano et al., 2003; Kaur et al., 2014). It is released to the rhizosphere by plants and has an inhibitory effect on root growth of a number of weeds (Fernández-Aparicio et al., 2013). Sarwar and Kremer (1995) showed that at a concentration of 10^-5^ M L-tryptophan alone had no effect on pigweed root growth but repressed pigweed root growth in presence of deleterious rhizobacteria. The effect of L-Tryptophan can be indirect as it can be metabolized to the plant phytohormone auxin by rhizosphere microorganisms and affect root growth in a species and dose-dependent manner (Sarwar and Kremer, 1995). Auxin can also increase lateral and adventitious rooting, resulting in an enhanced root exudation as well as mineral and nutrient uptake (Lambrecht et al., 2000; Mejri et al., 2010). L-tryptophan in the presence of plant growth promoting rhizobacteria had positive effects on wheat growth (Ul Hassan and Bano, 2015). The above-cited works do not explain the activity of BK-P root exudates on pigweed. At the present stage of our knowledge, we ignore the role of L-Tryptophan in the BK-P root interaction.

Buckwheat root exudates were already partially characterized in previous studies (Kalinova et al., 2007; Kato-Noguchi et al., 2007). Kalinova et al. (2007) showed inhibition of lettuce growth by soil from buckwheat stands at the branching and flowering stage and suggested that palmitic acid and gallic acid derivatives might be implicated in lettuce growth reduction. Gallic and palmitic acid are known to be released in root exudates and to be involved in plant growth regulation (Bertin et al., 2003; Latif et al., 2017). Additionally, in 60-days-old *Fagopyrum cymosum* plants, four fatty acids, including palmitic acid, were suggested to be allelochemicals of buckwheat (Tsuzuki et al., 1987). We did not find these compounds as statistically differentially abundant between our conditions.

## Conclusion

Allelopathic effects are usually not related to a single substance (Cheng and Cheng, 2015), but a mixture of allelochemicals. Therefore, we are considering the overall changes rather than to focus on single molecules. Characterization of root exudates produced in presence of heterospecific plants is a new approach to elucidate root interactions and interesting markers that have not been identified earlier in the literature might be discovered. The next step will be to identify which compounds in the complex root exudates of the BK-P condition are responsible for plant recognition and subsequent growth suppression. By subsequent chromatographic fractionation (Duke, 2015), it might be possible to assess if the whole bench of exudate chemicals is necessary for activity or only some specific fraction. Chromatographic purification of selected chemicals would also be useful for structure characterization by multidimensional nuclear magnetic resonance (NMR). This would lead to a better understanding of the root to root communication and the mechanism in plant–plant recognition, particularly crop-weed recognition. Furthermore, different varieties of buckwheat will be studied with regard to their root exudation profile and the associated rhizosphere microorganisms both in the laboratory and in the field within a crop rotation. This allows to elucidate whether the weed suppressive effect differs between varieties and how weed control in the field can be optimized.

## Author Contributions

AG, CE, and JW designed the research. AG, GG, CE, and CS conducted the research. AG and GG collected and analyzed the data. AG, GG, CS, and JW wrote the manuscript.

## Conflict of Interest Statement

The authors declare that the research was conducted in the absence of any commercial or financial relationships that could be construed as a potential conflict of interest.

## Abbreviations

BK: buckwheat
BK-P: buckwheat/pigweed mixed culture
C: control
I: direct root interactions
M: no direct root interactions, mesh bag
P: pigweed
